# New Concepts in Malaria Pathogenesis: The Role of the Renin-Angiotensin System

**DOI:** 10.3389/fcimb.2015.00103

**Published:** 2016-01-07

**Authors:** Leandro S. Silva, João Luiz Silva-Filho, Celso Caruso-Neves, Ana Acacia S. Pinheiro

**Affiliations:** ^1^Laboratório de Bioquímica e Sinalização Celular, Instituto de Biofísica Carlos Chagas Filho, Universidade Federal do Rio de JaneiroRio de Janeiro, Brazil; ^2^Instituto Nacional de Ciência e Tecnologia em Biologia e Bioimagem Conselho Nacional de Desenvolvimento Científico e Tecnológico/MCTRio de Janeiro, Brazil; ^3^Instituto Nacional para Pesquisa Translacional em Saúde e Ambiente na Região Amazônica Conselho Nacional de Desenvolvimento Científico e Tecnológico/MCTRio de Janeiro, Brazil

**Keywords:** malaria, invasion, erythrocytes, renin-angiotensin system, immune response, host-cell interaction, T cells, *Plasmodium falciparum*

## Abstract

Malaria is a worldwide health problem leading the death of millions of people. The disease is induced by different species of protozoa parasites from the genus *Plasmodium*. In humans, *Plasmodium falciparum* is the most dangerous species responsible for severe disease. Despite all efforts to establish the pathogenesis of malaria, it is far from being fully understood. In addition, resistance to existing drugs has developed in several strains and the development of new effective compounds to fight these parasites is a major issue. Recent discoveries indicate the potential role of the renin-angiotensin system (RAS) in malaria infection. Angiotensin receptors have not been described in the parasite genome, however several reports in the literature suggest a direct effect of angiotensin-derived peptides on different aspects of the host-parasite interaction. The aim of this review is to highlight new findings on the involvement of the RAS in parasite development and in the regulation of the host immune response in an attempt to expand our knowledge of the pathogenesis of this disease.

## Introduction

Malaria is a serious public health problem. The most severe form of the disease is related to *Plasmodium falciparum*, which is responsible for many deaths every year (World Health Organization, 2014). High parasitemia along with an impaired immune response are important elements responsible for the severity and lethality of the disease (Schofield and Grau, 2005).

Malaria-associated symptoms appear during the blood stage of the disease with infection of erythrocytes. Erythrocyte invasion by *Plasmodium* has always been credited to parasitic mechanisms and host-cell participation regarded as passive. However, new findings highlight the importance of host-specific signaling pathways that can control parasite invasion and development (Harrison et al., 2003; Murphy et al., 2006; Saraiva et al., 2011).

In this context, a multi-center study, based on genetic epidemiology, proposed an association between the occurrence of single nucleotide polymorphisms in the gene encoding the G alpha-S subunit and individual susceptibility to severe malaria, demonstrating that G-protein coupled receptor (GPCR) signaling in the host has an influence at the disease level (Auburn et al., 2008, 2010).

The renin-angiotensin system (RAS) is a proteolytic cascade that generates peptides that bind and signal through GPCRs. Classically, this system is involved in the regulation of intravascular volume and systemic blood pressure, acting in the renal and cardiovascular systems. In this peptidergic system, angiotensin II (Ang II) is formed from the enzymatic cleavage of angiotensinogen to angiotensin I (Ang I) by aspartyl protease renin, with subsequent conversion to Ang II by angiotensin-converting enzyme (ACE) (Mizuiri and Ohashi, 2015). Angiotensin-converting enzyme 2 (ACE2), a homolog carboxypeptidase of ACE can convert Ang II into angiotensin-(1–7) [Ang-(1–7)] or counter-regulate ACE activity competing for the same substrate, Ang I. Through cleavage of Ang I, ACE2 produces Ang-(1–9), which is converted to Ang-(1–7) by ACE (Ferrario, 2006). Therefore, the balance between ACE and ACE2 could determinate the levels of Ang II and Ang-(1–7).

Ang II exerts its actions via AT_1_ and AT_2_ receptors, which in principle, mediate opposite functions. AT_1_ receptors promote vasoconstriction, thirst and release of vasopressin and aldosterone, fibrosis, cellular growth, and migration (Fyhrquist and Saijonmaa, 2008). On the other hand, AT_2_ receptor stimulation leads to vasodilation, release of nitric oxide (NO), natriuresis, and inhibition of growth (Fyhrquist and Saijonmaa, 2008). Furthermore, the activity of both receptors may be altered by oligomerization, association with various interacting proteins, or ligand-independent effects (Villela et al., 2015).

Ang-(1–7) has its actions mediated specifically by the MAS receptor, inducing vasodilation by amplifying the effects of bradykinin, stimulating cGMP synthesis, and inhibiting the release of norepinephrine (Ferrario, 2006). Moreover, alamandine, another active hormone formed via decarboxylation of the aspartate radical group of Ang-(1–7), binds to the Mas-related receptor MrgD and has similar effects to Ang-(1–7) (Lautner et al., 2013). In this regard, ACE 2, Ang (1–7) and MAS receptors play a role counter-balancing excess activity of the Ang II/AT_1_ axis (Danilczyk and Penninger, 2006; Der Sarkissian et al., 2006). The expression and activity of ACE2 is upregulated by treatment with ACE inhibitors, such as captopril, promoting increased local production of Ang (1–7) (Ferrario, 2005; Ferrario et al., 2005).

The discovery of new RAS components and different local production has shifted attention to its non-classic effects. Here, we review recent findings that correlate local and systemic RAS with the host-parasite interaction in different levels of response. This could open new avenues in the elucidation of the molecular mechanisms involved in the pathogenesis of malaria.

## The role of RAS in erythrocyte invasion

Evidence in the literature showing that angiotensin II (Ang II) and related peptides impair parasite development were first demonstrated in the sexual cycle of *P*. *galinacium*, an avian malaria parasite (Maciel et al., 2008). The authors postulated that Ang II reduces the accumulation of sporozoites in the salivary gland of the mosquito vector by directly disturbing the parasite membrane. However, this effect seems to occur in a receptor-independent manner.

Other studies have reported an apparent protective effect of Ang II in malaria. A genetic association study was carried out in Orissa, India, to search for a possible influence of polymorphisms in angiotensin I-converting enzyme (ACE) and angiotensin II-converting enzyme (ACE2) on the outcome of malaria. It was shown that the D allele of ACE I/D polymorphism, which increases Ang II production, is associated with mild malaria. ACE2 C → T substitution, a polymorphism that reduces ACE2 expression in the presence of the T allele also results in an increase in Ang II, by reducing its conversion to Ang-(1–7) (Dhangadamajhi et al., 2010). Further evidence that ACE and ACE2 polymorphisms and consequent increased plasma levels of Ang II are associated with malaria severity was demonstrated in people with an African genetic background (Gallego-Delgado and Rodriguez, 2014). In this population, it seems that these polymorphisms confer protection from severe malaria in childhood but deleterious effects, such as hypertension, in adulthood (Gallego-Delgado and Rodriguez, 2014). Thus, it was suggested that higher levels of Ang II protect against cerebral malaria. However, the molecular mechanism involved in disease protection was not shown.

Our group was the first to further characterize the molecular mechanisms involved in the anti-malarial effects of angiotensin peptides during the blood stage of *P. falciparum* (Saraiva et al., 2011). In accordance with previous studies, we found that Ang II decreased the invasion of human erythrocytes by *P*. *falciparum* in a dose-dependent manner. Even though Ang II receptors, AT_1_ and AT_2_, were demonstrated in the erythrocyte membrane, surprisingly, this effect was not mediated by these receptors. This evidence suggested the metabolism of Ang II and the generation of other biologically active peptides. Mas receptor expression in the erythrocyte membrane and its ligand, Ang-(1–7), in the culture supernatant were detected. Ang-(1–7) reduced erythrocyte invasion by the same magnitude as Ang II. This effect was sensitive to A779, a Mas antagonist and not blocked by Losartan, an AT1 receptor antagonist.

**Figure 1:**
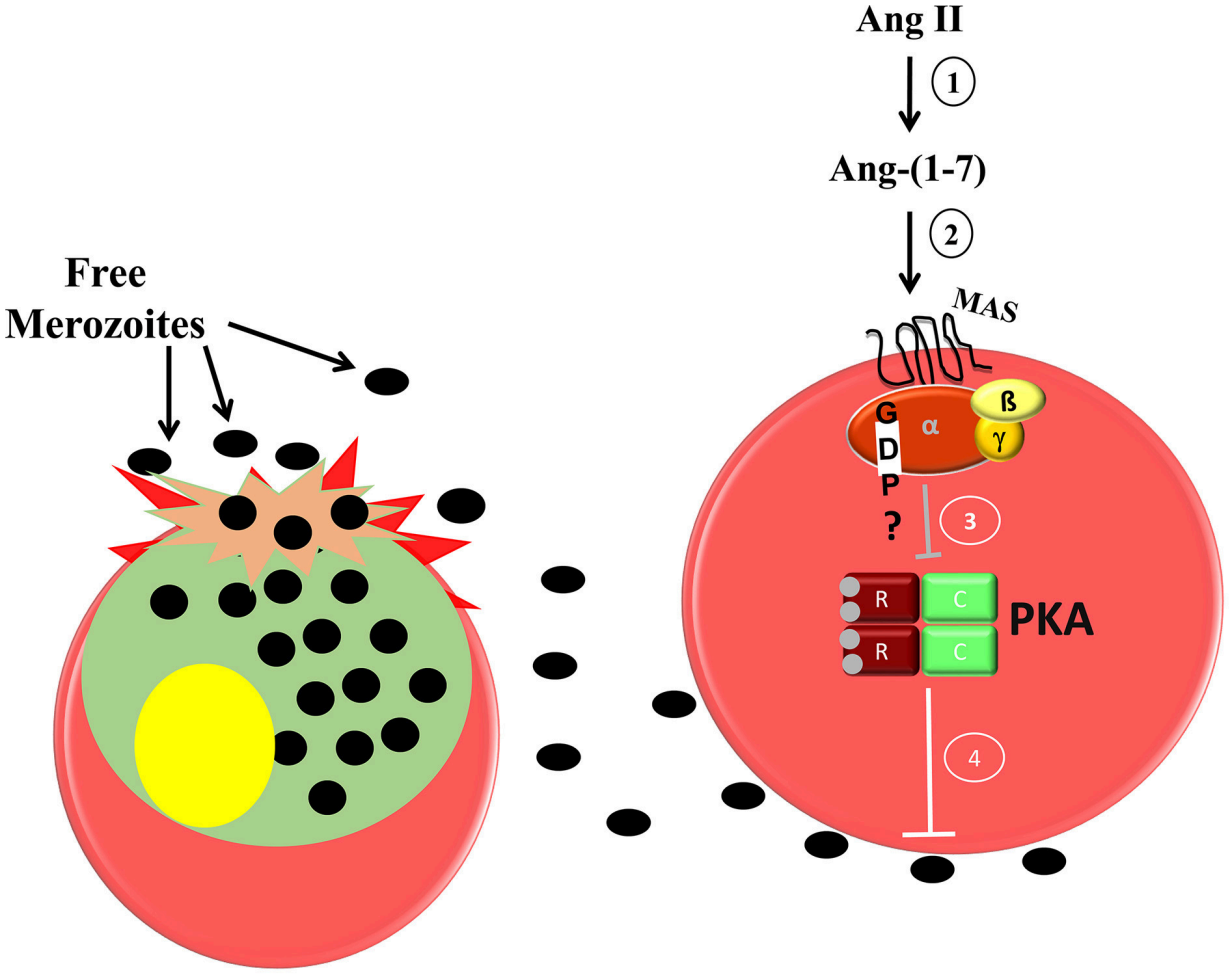
**Proposed model of the inhibitory effect of Ang-(1–7) during *P. falciparum* invasion of human erythrocytes**. 1, Ang II is converted to Ang-(1–7) by ACE 2 activity; 2, Ang-(1–7) binds its specific receptor; Mas, which is expressed in the human erythrocyte membrane; 3, a signaling pathway is triggered inhibiting PKA activity; 4, decreased PKA activity impairs merozoite invasion in the erythrocytes.

In erythrocytes, the increase in cAMP levels and consequent PKA activation improve erythrocyte invasion by *P. falciparum* (Harrison et al., 2003). Accordingly, cAMP analog increases erythrocyte invasion *in vitro*, whereas PKA inhibitor reduces it and reverses the stimulation induced by the cAMP analog. Moreover, PKA activity is reduced in the presence of Ang-(1–7) in a Mas-dependent manner (Saraiva et al., 2011). Our data suggest that Ang II is metabolized to Ang-(1–7), which binds specifically to the Mas receptor, decreasing PKA activity and consequently reducing parasite invasion (Figure 1).

Interestingly, (Gallego-Delgado et al., 2015) demonstrated a significant reduction in blood parasitemia in mice infected with *Plasmodium berghei* ANKA treated with a supraphysiological concentration of Ang II. They also observed delayed establishment of cerebral malaria and decreased incidence of brain hemorrhage followed by a modest increase in survival. Because Ang II is quickly degraded forming different peptides, the improvement in the condition of mice could be attributed to increased levels of plasma Ang-(1–7), corroborating with our evidence.

Because angiotensin peptides have biological effects on the host, their use in anti-plasmodium therapy *in vivo* could have some limitations. Recent studies have proposed structural changes in RAS peptides, aiming to block their vascular and renal effects without affecting the anti-plasmodial action (Maciel et al., 2008; Chamlian et al., 2013; Silva et al., 2014, 2015). Different angiotensin-restricted analogs have been synthesized and tested for their anti-plasmodial effects on *P. falciparum* and *P. gallinaceum* (Chamlian et al., 2013; Silva et al., 2014, 2015; Torres et al., 2015) and some have been shown to have a potential anti-plasmodial effect.

It is possible to assume that angiotensin peptides inhibit the sexual and blood stage of avian as well as human parasites. However, malaria is a multi-factorial disease and as well as the increase in parasitaemia, other parasite-host interactions are important in the pathogenesis of the disease. The syndrome is marked by intense inflammatory immune responses, sequestration of leukocytes, and parasitized erythrocytes in the microvasculature. Involvement of angiotensin peptides in the regulation of immune system has been suggested (Iwai et al., 1996; Nataraj et al., 1999; Bush et al., 2000; Jurewicz et al., 2007; Hoch et al., 2008; Geara et al., 2009; Silva-Filho et al., 2011, 2013, 2015). In the following, the role of RAS in the modulation of T-cell responses during infection is reviewed.

## T-cell regulation by the Ang II/AT_1_ receptor axis in malaria

In recent years, Ang II has been found to be a pro-inflammatory molecule and pro-fibrotic agent, inducing ROS production, cell growth, apoptosis, cell migration, and differentiation, which contribute to progressive damage to organ function in disease (Iwai et al., 1996; Nataraj et al., 1999; Bush et al., 2000; Donadelli et al., 2000; Ruster and Wolf, 2006; Guzik et al., 2007; Jurewicz et al., 2007; Hoch et al., 2008; Geara et al., 2009; Silva-Filho et al., 2011, 2013, 2015).

With regard to lymphoid tissue, our group and others have described the expression of all RAS components in T cells (Nataraj et al., 1999; Jurewicz et al., 2007; Hoch et al., 2008; Silva-Filho et al., 2011, 2013, 2015). T cells possess the necessary enzymatic machinery to convert angiotensinogen substrate to active Ang II in sufficient quantities to induce cellular autocrine and/or paracrine effects, pointing out Ang II as a co-stimulatory molecule for T-cell functions, such as activation, migration, differentiation, and cytokine production (Nataraj et al., 1999; Jurewicz et al., 2007; Hoch et al., 2008; Silva-Filho et al., 2011, 2013, 2015). Ang II levels produced by T cells seem to be subtype and tissue specific, because CD4^+^ T cells produce higher amounts of Ang II than CD8^+^ T cells and blood-derived T cells produce more Ang II than cells harvested from the spleen (Jurewicz et al., 2007; Hoch et al., 2008). Similarly, CD4^+^ T cells express slightly higher levels of AT_1_ receptor compared with CD8^+^ T cells, whereas AT_2_ receptor was barely detected in these cells either on the surface or intracellularly (Jurewicz et al., 2007; Hoch et al., 2008). In addition, activated T cells, *in vitro* by anti-CD3 or *in vivo* by *Plasmodium* antigens, showed upregulated AT_1_R expression while AT_2_R expression was not changed (Silva-Filho et al., 2011, 2013).

An experimental model with *P. berghei* ANKA has been a useful tool to understand the pathogenesis of malaria, including cerebral malaria, one of the most severe clinical forms of *P. falciparum* infection. Using murine infection, this model is well validated to mimic human disease in some key pathologic aspects (Schofield and Grau, 2005). Different effector cells such as CD4^+^ T cells, CD8^+^ T cells, natural killer T cells, and natural killer cells also contribute to the pathogenesis of the disease (Yañez et al., 1996; Belnoue et al., 2002; Hansen et al., 2003; Schofield and Grau, 2005; Nie et al., 2009; Ohayon et al., 2010). However, T cells are the one of the most important effector cells involved in the syndrome after infection with *P. berghei* (Yañez et al., 1996; Belnoue et al., 2002; Schofield and Grau, 2005; Nie et al., 2009; Ohayon et al., 2010), because intravascular infiltration of T cells in the brain vasculature during infection could be associated with a local inflammatory response, determining the severity of the disease (Yañez et al., 1996; Belnoue et al., 2002; Hansen et al., 2003; Schofield and Grau, 2005; Schofield, 2007; Nie et al., 2009; Ohayon et al., 2010). Thus, the signals and consequently the cellular and molecular mechanisms regulating the T-cell immune response are crucial to understand the role of these cells during infection.

**Figure 2:**
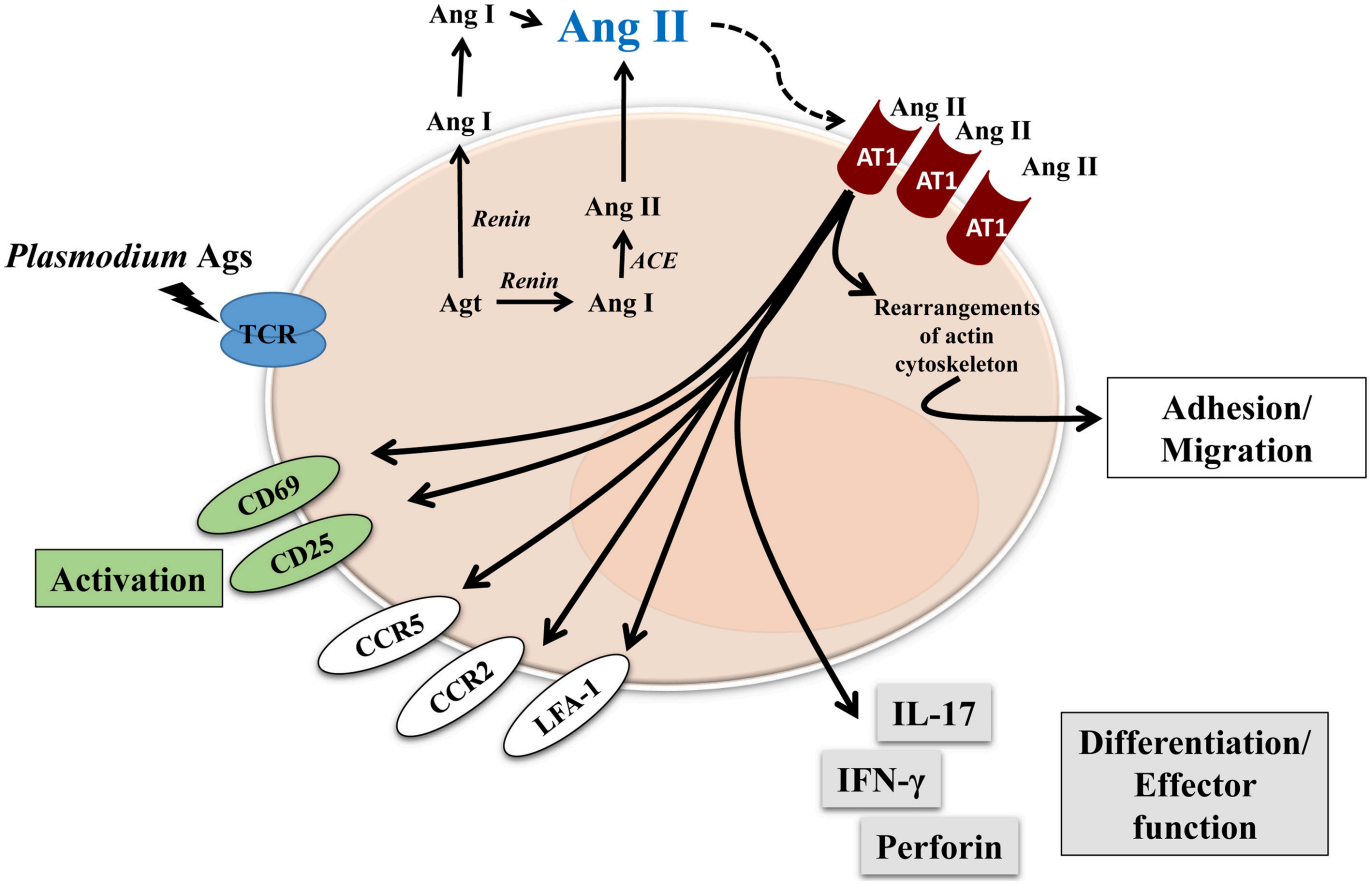
**Proposed model of Ang II effect on T cells during blood-stage of *Plasmodium* infection**. Renin-Angiotensin System and Angiotensin receptors are expressed by T cells. Endogenous Ang II could induce autocrine or paracrine effects, mediated by AT_1_R, which is upregulated during *Plasmodium* infection. Different effects have been characterized, such as: activation, observed by upregulation of CD69 and CD25; adhesion/migration, mediated by upregulation of chemokine receptors CCR2 and CCR5, adhesion molecule LFA-1 as well as rearrangements of actin cytoskeleton. Finally, Ang II, via AT_1_R, is also involved in T-cell differentiation/effector function acquisition observed by increased production of cytokines IL-17 and IFN-γ, and perforin, a marker of cytotoxic activity.

So far, the effect of Ang II and its receptors on T cells has been well described using *in vitro* systems and *in vivo* models, most of them exploring renal or cardiovascular diseases, but it is not clear how RAS components influence the T-cell response during parasitic diseases. In this regard, we found that Ang II, via the AT_1_ receptor, was an important mediator for T-cell activation, migration and adhesion during *P. berghei* ANKA infection in mice (Silva-Filho et al., 2013). We showed that the RAS could be involved in the pathogenesis of not only non-infectious diseases but also parasitic disease. Our results observed in adhesion/migration experiments with splenic T cells from *P. berghei* ANKA-infected mice confirmed observations from other studies (Figure 2) (Bush et al., 2000; Donadelli et al., 2000; Jurewicz et al., 2007; Crowley et al., 2008; Silva-Filho et al., 2011, 2013, 2015).

Endogenously produced Ang II induces T-cell adhesion in endothelial cells and migration via the AT_1_ receptor (Figure 2; Jurewicz et al., 2007; Silva-Filho et al., 2011, 2013, 2015). As a possible cellular mechanism, Ang II upregulated LFA-1, CCR2, and CCR5 expression in T cells (Figure 2). According to previous observations (Crowley et al., 2008), Ang II also induces cytoskeleton rearrangement and cell spreading, via the AT_1_ receptor, when T cells are stimulated to adhere *in vitro* (Silva-Filho et al., 2013). These results explain the role of Ang II/AT_1_ receptor in inducing T-cell adhesion/migration *in vitro* and reduced sequestration of total CD3^+^ and CD8^+^ T lymphocytes in the brain of mice infected with *P. berghei* ANKA when treated with captopril, an ACE inhibitor (Figure 2) (Silva-Filho et al., 2013). It seems that the Ang II/AT_1_ receptor axis drives activation followed by sequestration of T lymphocytes in the brain of infected animals (Silva-Filho et al., 2013).

In addition to decreased T-cell sequestration in the brain, inhibition of Ang II/AT_1_ receptor signaling using losartan or captopril in *P. berghei* ANKA-infected mice promoted some survival benefits and attenuation of signals involved in the development of cerebral malaria, such as cerebral oedema (Silva-Filho et al., 2013). Reduced blood parasitaemia in treated mice up to 5 days post infection was also observed. It is well known that captopril increases the levels of Ang(1–7). As described above, Ang(1–7) reduces *P. falciparum* invasion *in vitro* (Silva et al., 2014). Similarly captopril-treated mice infected with *P. berghei* ANKA showed lower parasitaemia than control mice throughout the experiments (Silva-Filho et al., 2013). These results corroborate the idea of GPCR agonists, such as Ang(1–7), regulating parasite invasion (Saraiva et al., 2011). Therefore, modulation of the activity of RAS components could be another factor in the pathogenesis of malaria.

## Targeting RAS is a possible therapeutic strategy?

Together, these observations show that modulation of the activity of RAS components could be considered as another factor in the pathogenesis of malaria. The pharmacological approach used by us and others initially aimed to prove the influence of RAS in malaria pathogenesis and emerged as an interesting target in a therapeutic strategy.

Although this matter is not clear, one could suggest that the protective effect of Ang II on parasite invasion and cerebral malaria seems to be a result of its conversion to Ang (1–7) and its binding to MAS receptors. Our results, schematically represented in Figures 1, 2, clearly indicate that by promoting Ang II conversion to Ang (1–7) by captopril treatment, Ang (1–7) stimulates MAS receptors on erythrocytes, inhibiting PKA activity with a consequent decrease in parasite invasion and growth (Figure 1). At same time, decreased AT_1_R stimulation on T lymphocytes by blocking receptors with losartan or by decreasing Ang II levels with captopril reduces the exacerbated and harmful pro-inflammatory response mediated by these cells (Figure 2). These effects on both parasitemia and T-cell response seem to improve disease outcome. Indeed, as discussed earlier, Silva-Filho et al. (2013) observed that both captopril (an ACE inhibitor) and losartan (an AT_1_R antagonist) treatments reduced parasitemia in mice infected with *P. berghei* ANKA, probably by increasing Ang (1–7) levels, and reduced T-cell activation, adhesion, migration, and sequestration in the brain by inhibiting AT1R stimulation in these cells.

Therefore, we could suggest that targeting RAS using agents that promote the conversion of Ang II to Ang (1–7), favoring Mas signaling over AT_1_R signaling in erythrocytes and at same time inhibiting AT_1_R stimulation in immune cells, is an interesting approach to a potential therapeutic strategy.

## Conclusions

The studies and results described in this review shed some light on the functions and sources of RAS components. As already observed in renal and cardiovascular diseases, unbalanced RAS activity can also contribute to the pathogenesis of parasitic infections via pathophysiologic actions. The work described in this review may lead to new possibilities for therapeutic interventions.

## Author contributions

LSS, JLS, CCN, and AAP conceived the idea and wrote the first draft of the manuscript. All authors read and approved the final version.

## Funding

The research papers from the group used to prepare this minireview received grants from: Conselho Nacional de Desenvolvimento Científico e Tecnológico—CNPq [471801/2010-0]; and Fundação Carlos Chagas Filho de Amparo à Pesquisa do Estado do Rio de Janeiro—FAPERJ [Grant Numbers E-26/110.085/ 2014, E-26/102.170/2013, E-26/110.605/2012, E-26/ 101.450/2010, E-26/110.792/2009].

### Conflict of interest statement

The authors declare that the research was conducted in the absence of any commercial or financial relationships that could be construed as a potential conflict of interest. The reviewer Salah Mécheri and handling Editor declared their shared affiliation, and the handling Editor states that the process nevertheless met the standards of a fair and objective review.
